# Adnexal torsion in pregnancy after in vitro fertilization: Case report and literature review

**DOI:** 10.1097/MD.0000000000024009

**Published:** 2021-01-22

**Authors:** Meiling Yu, Yanhong Liu, Dongyun Jia, Tian Tian, Qi Xi

**Affiliations:** Center for Reproductive Medicine and Center for Prenatal Diagnosis, First Hospital, Jilin University, 71 Xinmin Street, Chaoyang District, Changchun, Jilin Province, China.

**Keywords:** adnexal torsion, pregnant, in vitro fertilization, detorsion, outcome

## Abstract

**Rationale:**

Torsion is the most common gynecologic emergency of the adnexal mass occurring during pregnancy. We report the clinical data of a case of twin pregnancy with adnexal torsion after in vitro fertilization (IVF) and embryo transfer, in which the patient underwent surgery for adnexal detorsion and experienced preterm delivery. One child died as a neonate, and the other child was diagnosed with hematological disease, which, to our knowledge, has not been reported previously. We also performed a systematic literature review to increase knowledge of the need for prompt surgical intervention.

**Patient concerns:**

The patient was a 32-year-old pregnant woman, who presented to our center with acute onset of stabbing and non-radiating continuous lower left abdominal pain after urination, of 6 hours duration.

**Diagnosis:**

Physical examination revealed that the lower abdominal tenderness was worse on the left side, and there were no signs of peritonitis. Transvaginal ultrasonographic examination indicated a multiloculated left ovary measuring 12.1 × 7.1 cm with sparse blood flow. The size of the largest cyst was 7.2 × 6.6 cm, the right ovary appeared normal, and two live fetuses were seen.

**Interventions:**

laparoscopy was performed 1.5 hours later, which revealed a cystic and multilocular left ovary with a black purplish surface and thin wall. The left ovary and left fallopian tube had undergone 720° torsion (3 rotations), and detorsion was performed laparoscopy.

**Outcomes:**

The left adnexa recovered to near normal appearance 20 minutes postoperatively. The patient was discharged from hospital 5 days postsurgery, without complications. Unfortunately, the patient delivered two preterm babies at 30 weeks of gestation.

**Conclusions:**

We should be alert to the possible risk of adnexal torsion in pregnant women after IVF. Adnexal torsion necessitates prompt surgical intervention, detorsion and preserving ovarian function are the main treatment methods. Furthermore, the possibility of recurrence, and pregnancy outcomes for the patient, as well as newborn health, should be considered.

## 1 Introduction

Adnexal torsion is a high-risk situation for patients, presenting as acute abdominal pain during pregnancy, and it is a serious complication of assisted reproductive therapy (ART). With an estimated incidence of 1 in 5000 natural pregnancies,^[1]^ the incidence increases to 0.1% after ART,^[2]^ and has been reported to be as high as approximately 6% to 16% in in pregnant women with ovarian hyperstimulation syndrome (OHSS).^[3,4]^ Twin pregnancies are 3.5 times more likely to involve adnexal torsion than single pregnancies.^[4]^ with increased numbers of infertile women undergoing ART, it is essential to increase awareness of adnexal torsion, in women who become pregnant by this method, and to highlight the clinical characteristics, therapy, and pregnancy outcomes. We report the clinical data of a case of twin pregnancy with adnexal torsion after IVF and embryo transfer, and review the related literature in detail.

## 2 Case report

**Figure 1:**
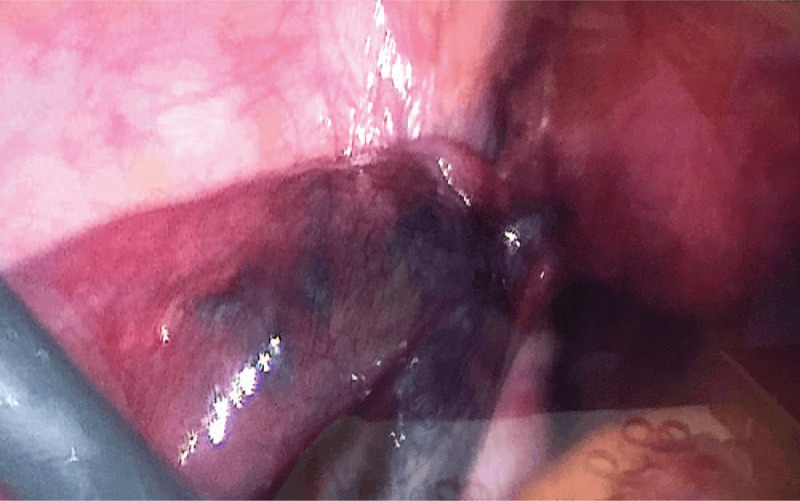
Detorsion of left ovary and left fallopian tube.

**Figure 2:**
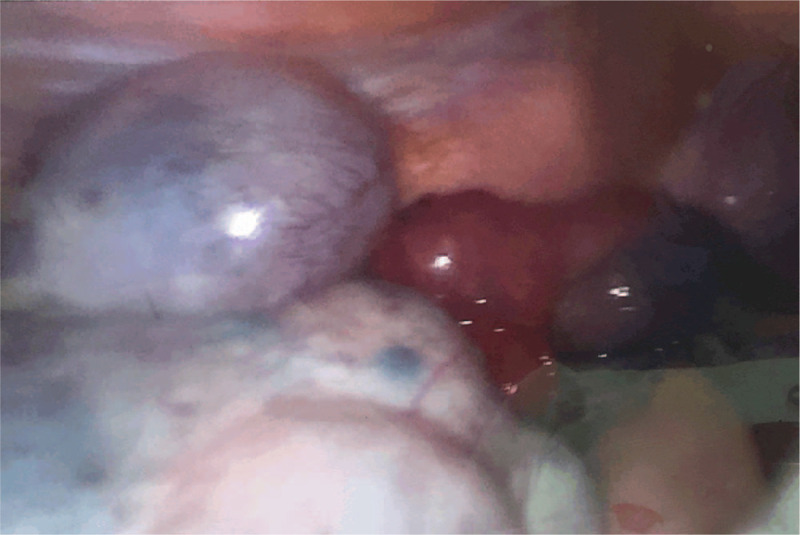
20 Twenty minutes after detorsion of left ovary and left fallopian tube.

**Figure 3:**
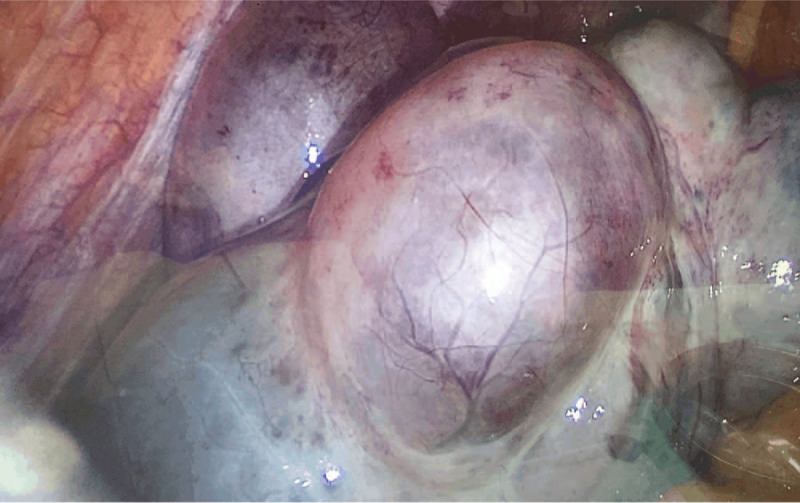
Twenty minutes after detorsion of left ovary.

The patient was a 32-year-old pregnant woman, who presented to our center for acute onset of stabbing and nonradiating continuous lower left abdominal pain after urination, of 6 hours duration. She had no history of prior abdominal trauma or surgery. Her husband had a diagnosis of male factor infertility. After ovulation induction, she underwent IVF with the gonadotropin-releasing hormone (GnRH) antagonist protocol. Twelve days later, 11 oocytes and 3 embryos were obtained. On day 3, ultrasonography showed that the right ovary measured 4.7 × 3.5 cm, the left ovary measured 6.6 × 6.0 cm, and the maximum depth of the recto-uterine pouch free fluid was 3.9 × 2.3 cm. Two fresh embryos were transferred and this was followed by a 60 mg progesterone injection once a day, and oral dydrogesterone 10 mg twice a day. On day 30 after embryo transfer, transvaginal ultrasonography identified intrauterine twin pregnancy, and the left ovary measured 13.2 × 13.1 cm. The relatively large anechoic rim sizes were 7.1 × 5.8 cm, 7.6 × 5.6 cm, and 7.3 × 5.1 cm, and the posterior fornix fluid measured 5.2 × 2.6 cm. The outpatient doctor suggested the patient should avoid strenuous activities. On day 45 after embryo transfer, the size of the left ovary was 13.8 × 9.5 cm. Six hours before seeking treatment, the patient experienced sudden onset of severe progressive abdominal pain after urination, and pain on the left lower side was obvious. She could not obtain relief form the pain after changing position, and the pain was accompanied by nausea, vomiting. Physical examination revealed that the lower abdominal tenderness was worse on the left side, and there were no signs of peritonitis. Transvaginal ultrasonography indicated a multiloculated left ovary measuring 12.1 × 7.1 cm with sparse blood flow; the size of the largest cyst was 7.2 × 6.6 cm, the right ovary appeared normal, and the two live fetuses were seen. Laboratory blood examination revealed increased white blood cell count (10,140 cells/μl), neutrophil granulocyte percentage (92%), and cancer antigen 125 (CA125) concentration (196.9 U/ml). Laparoscopy was performed 1.5 hours later, which revealed that the left ovary was cystic and multilocular with a black purplish surface and thin wall. The left ovary and left fallopian tube had undergone 720° torsion (3 rotations), and detorsion was performed laparoscopy (Fig. 1). The largest cyst in the left ovary was ruptured, and light-yellow fluid was seen during the operation. The left adnexa recovered to near normal appearance 20 minutes after detorsion (Fig. 2 and Fig. 3). One day after the operation, blood laboratory examination revealed the following: CA125: (177.7 U/ml), white blood cell count: (7940 cells/μl), and neutrophil granulocyte percentage: (75%). One month after the operation, the CA125 concentration was 43.42 U/ml, and 3 months after the operation, transabdominal ultrasonography showed that bilateral adnexa were normal. Unfortunately, the patient delivered two preterm babies at 30 weeks of gestation. One child died on the second day after delivery, and the other child was diagnosed with diffuse large B-cell lymphoma 2 months after delivery. This child underwent enterectomy and enterostomy for necrotizing enterocolitis, and received chemotherapy, now he is in fellow up.

## 3 Discussion

Adnexal torsion is the most common gynecologic emergency of an adnexal mass occurring during pregnancy, and it can occur at any age.^[5]^ Recently, the incidence has increased, which is attributed to the increase in pregnancies resulting from IVF.^[1,2]^ Reportedly, 60% (18/30) of cases occur between the 10th and 17th weeks of gestation, compared with 36.7% (11/30) occurring during the 15th and 16th weeks of gestation. In addition, the incidence decreases to <5.9%, if torsion does not occur before the gestational age of 20 weeks.^[9]^ The right adnexa is most commonly involved,^[6–8]^ and the mass diameter in adnexal torsion events is often 6 to 10 cm^[6,9,10]^ (Table 1).

**Table 1:** Adnexal torsion in pregnant after in IVF cases reports.

Author and year	Case	Age (years)	Number of transferred embryos	Pregnancy	Weeks of pregnancy	Side	Maximal size(cm)
Seiji et al, 2019^[7]^	1	40	–	Singleton	6w	R	6.9
Kim et al, 2017^[8]^	1	29	–	Singleton	11w	R	8
Hsing-chun et al, 2015^[6]^	2	30.5 (M)	3	TP (1/2), Singleton (1/2)	7w (M)	R, R	9 (M)
Turgut et al, 2014^[22]^	1	28	2	Twin fetuses	23w	R	6 (mass)
Dursun et al, 2013^[19]^	1	–	–	Two fetuses	25w	L	8
Spitzer et al, 2012^[10]^	5	33 (M)	1 (3/5), 2 (2/5)	Singleton (5/5)	7.8w (M)	L (1/5), R (4/5)	8.7 (M)
Giulini et al, 2010^[16]^	1	31	3	Singleton	Two days after ET	R	10
Hasiakos et al, 2008^[1]^	2	33	–	Two fetuses (1/2), Singleton (1/2)	10w (M)	R, R	10.3 (M)
Weitzman et al, 2008^[32]^	1	32	2	Singleton	L (two days after ET),		
R (6w), R (7w)	L, R, R	7.3 (M)					
Arena et al, 2009^[33]^	1	38	3	Two fetuses	10w	R	7.9
Rackow et al, 2007^[34]^	2	28	3	Singleton	R (7w), L (9w)	R, L	9 (M)
Djavadian et al, 2004^[17]^	1	29	–	Singleton	R (7w), R (13w), L (15w)	R, R, L	8
Gorkemli et al, 2002^[35]^	5	31.6 (M)	–	–	7w (M)	–	8 (M)
Chew et al, 2001^[15]^	1	38	3	Singleton	6w	L	8.6
Anil et al, 2001^[18]^	1	39	3	Singleton	1w after ET	L	–
Mashiach et al, 2001^[12]^	12	31.2	–	–	10.3w (M)	L (7/12). R (5/12)	12.9 (M)

All patients experience sudden onset of pain, and most patients experience nausea and vomiting. The viability of an ovary gradually decreases with longer torsion time.^[11]^ Emergency operation should be performed to avoid ovarian damage, if the patient's symptoms, physical examination, and auxiliary examination suggest adnexal torsion, especially if the patient is pregnant. Rousseau et al^[11]^ reported that the ten ovarian torsion operated within 24 hours, 80% had conservative ovarian surgery; the 15 ovarian torsion operated between 24 and 72 hours form the onset of pain, 47% had conservative ovarian surgery; and to only 9% if surgery is performed at >72 hours. The time between the onset of symptoms in our patient and admission to hospital was 6 hours, and adnexal detorsion was performed 3 hours later. Mashiach et al^[12]^ concluded that physicians should warn pregnant patients of the increase risks associated with the following four conditions:

1. human menopausal gonadotropin (HMG) treatment;
2. enlarged, hyperstimulated ovaries;
3. pregnancy; and
4. adnexal torsion.

A corpus luteum cyst is the second most frequent pathology associated with adnexal torsion, with 33.3% (6/18) of patients with this pathology developing torsion.^[9]^ Oelsner et al reported that the most frequent histology in ovarian cystectomy is a corpus luteum cyst (12/31); 58% (18/31) of removed cysts are functional.^[13]^ In 1946, Way^[14]^ reported the first conservative management of adnexal torsion. In the report, adnexal structures were detorsed in 15 cases, and patients underwent cystectomy. Recently, detorsion via laparoscopy has become nearly standard treatment.^[15–18]^ Some reports indicate that adnexal torsion can be treated by laparoendoscopic single-site surgery or transvaginal ovarian cystectomy during pregnancy at 25 to 27 weeks.^[19,20]^ Although recurrence of adnexal torsion with conservative management is rare, it can threaten ovarian function and fetal safety.

We performed a systematic literature review to identify case reports of adnexal torsion during pregnancy after IVF. We identified 38 cases; 2 of these cases developed adnexal torsion recurrence, and both underwent three operations. Djavadian et al^[17]^ suggested that detorsion should be performed as soon as clinical symptoms appear in cases of recurrence, and oophoropexy is strongly recommended to avoid recurrent torsion. Recurrent adnexal torsion can also be prevented by adnexal detorsion and shortening the utero-ovarian ligament.^[15,21]^ Seven cases of ovarian torsion were reported by Hosny et al^[22]^; 2/7 cases had OHSS, and 50% of the patients showed clinical symptoms and hemorrhagic cysts. Fortunately, there was no recurrence in these 2 patients, who underwent detorsion and oophoropexy. Based on the above cases and investigations, it can be concluded that detorsion and oophoropexy can be performed easily and quickly and this approach is more appropriate for ovarian torsion in pregnant women and OHSS patients.

According to the related literature, most cases suggest that surgical treatment is beneficial to treat adnexal torsion^[6–8,19,23]^ (Table 2). In 40 cases of adnexal detorsion reported by Oelsner et al,^[24]^ 13 patients were treated only with detorsion, 12 patients were treated with detorsion and cyst aspiration, and 15 patients underwent detorsion and cystectomy, with an average follow-up time of 4.07 years, ultrasonography showed follicular development in 35/37 cases. Adnexal infarction is considered an uncommon symptom, but is a serious complication for conservative surgical management of adnexal torsion. Adnexal infarction was first reported by Ruth et al,^[25]^ in the report, laparoscopic cyst drainage and adnexal detorsion was perform intraoperatively, and right adnexectomy was performed 40 hours after the first procedure for the postoperative complication of adnexal infarction; however, the authors did not discuss the infant-related outcomes. Wiser et al^[4]^ reported 10 cases of adnexal torsion in 165 IVF patients with OHSS in the first trimester who underwent laparoscopic treatment under general anesthesia. Seven twins and three singletons were delivered successfully, and the patients with OHSS and ovarian torsion experienced uneventful postoperative courses, and delivered normal babies at term. However, when infants are delivered prematurely, our patient outcomes may be worse. One of the infants in our report died 1 day after birth, and the other was diagnosed with necrotizing enterocolitis and non-Hodgkin lymphoma. The relationship between surgery for adnexal torsion and adverse pregnancy outcomes is not currently understood. Whitecar et al^[26]^ considered that patients undergoing laparotomy after 23 weeks of gestation have significantly higher adverse pregnancy outcome than patients undergoing laparotomy earlier in gestation. In contrast, another report showed that 3/60 infants were delivered after 22 weeks’ gestation, and then died perinatally,^[27]^ Two of the three deaths were attributed to 18-trisomy and multiple anomalies incompatible with life, the authors suspected that surgical intervention at <24 weeks of gestation per se might not have been responsible for the adverse outcomes. It is still controversial whether children conceived by ART have an increased risk of developing leukemia and Hodgkin lymphoma.^[28,29]^ However, recent studies showed that laparoscopy for adnexal detorsion in pregnancy may be associated with better outcomes for the patient, and perinatally, for the infant.^[6–8,19,23]^ Thirty eight cases were identified in our systematic reviewed literature, 27/29 cases (93.1%) delivered healthy infants. To our knowledge, our case is the first report of a pregnant patient undergoing surgery for adnexal detorsion and experiencing preterm delivery with neonatal death in 1 baby and hematological disease in the other. With our patient, we carefully considered that anesthesia-related drugs for the fetus were able to be used during pregnancy, as for all pregnant women. Preterm birth is the most frequent cause of neonatal death, and is also the second largest direct cause of death in children younger than 5 years of age, worldwide.^[30]^ Furthermore, multiple factors imply that using ART increases the rate of preterm birth.^[31]^

**Table 2:** Adnexal torsion in pregnant after IVF cases reports.

Author and year	Case	Age (years)	Operative procedure	Outcome of pregnancy	Outcome of fetal
Seiji et al, 2019^[7]^	1	40	Detorsion (LSC)	Delivery (38w)	Healthy infant
Kim et al, 2017^[8]^	1	29	Adnexectomy (LAP)	–	–
Hsing-chun et al, 2015^[6]^	2	30.5 (M)	Oophorectomy (LSC), Adnexectomy (LSC)	Ongoing (all)	–
Turgut et al, 2014^[22]^	1	28	Detorsion (LSC)	Cesarean section (35w)	Healthy infants
Dursun et al, 2013^[19]^	1	–	Adnexectomy (LESS)	Cesarean section (32w)	healthy infants
Spitzer et al, 2012^[10]^	5	33			
(M)	Detorsion and cyst puncture (LSC) (2/5), Detorsion (LSC) (3/5)	Vaginal delivery (3/5),missed abortion (1/5) (10w), induced abortion (1/5) (19w)	Healthy infant		
(3/5)					
Giulini et al, 2010^[16]^	1	31	Detorsion (LSC)	Delivery (40w)	Healthy infant
Hasiakos et al, 2008^[1]^	2	33	Adnexectomy (LSC), Detorsion	Cesarean section (37w), Delivery (39w)	Healthy infant
Weitzman et al, 2008^[32]^	1	32	Detorsion (LSC), Detorsion and cyst puncture (LSC)		
Detorsion and shortening ligament (LSC)	Vaginal delivery(38w)	Healthy infant			
Arena et al, 2009^[33]^	1	38	Adnexectomy (LSC)	Cesarean section	healthy infants
Rackow et al, 2007^[34]^	2	28	Detorsion and cyst puncture (LSC), Adnexectomy (LAP)	Vaginal delivery(38w)	Healthy infant
Djavadian et al, 2004^[17]^	1	29	Detorsion (LSC), Detorsion (LSC)		
Detorsion and shortening ligamentum (LSC)	Delivery				
(39w)	Healthy infant				
Gorkemli et al, 2002^[35]^	5	31.6 (M)	Detorsion or detorsion and cyst aspiration LSC(4/5), LAP(1/5)	Delivery	Healthy infant
Chew et al, 2001^[15]^	1	38	Detorsion (LSC)	Vaginal delivery (39w)	Healthy infant
Anil et al, 2001^[18]^	1	39	Detorsion (LSC)	Cesarean section (at term)	Healthy infant
Mashiach et al, 2001^[12]^	12	31.2 (M)	Detorsion(5/12), Adnexectomy (1/12), Detorsion and cystectomy (2/12), Detorsion and cyst puncture or aspiration (4/12)	Delivery (7/12), Abortion (2/12), Delivered prematurely (1/12), Ongoing (2/12)	–

In conclusion, adnexal torsion is a rare but acute complication that generally occurs in pregnant patients after IVF. With the increased use of ART, we should be alert to the possible risk of adnexal torsion in pregnant women after IVF, detorsion and preserving ovarian function were the main treatments. However, the following concerns must also be considered: ovarian function, preterm birth, neonatal death, and the health of the child. Therefore, large clinical studies are still required.

## Acknowledgments

We grateful acknowledge the support and assistance provide by all staff in The First Hospital’ Reproductive Medicine Center and thank the editors and reviewers for their help with this manuscript. We thank Jane Charbonneau, DVM, from Edanz Group (https://en-author-services.edanzgroup.com/) for editing a draft of this manuscript.

## Author contributions

**Methodology:** Yanhong Liu, Dongyun Jia.

**Supervision:** Tian Tian.

**Original draft:** Meiling Yu, Qi Xi.

**Writing – review and editing:** Meiling Yu, Qi Xi.
